# Low-cost sensor-integrated 3D-printed personalized prosthetic hands for children with amniotic band syndrome: A case study in sensing pressure distribution on an anatomical human-machine interface (AHMI) using 3D-printed conformal electrode arrays

**DOI:** 10.1371/journal.pone.0214120

**Published:** 2019-03-28

**Authors:** Yuxin Tong, Ezgi Kucukdeger, Justin Halper, Ellen Cesewski, Elena Karakozoff, Alexander P. Haring, David McIlvain, Manjot Singh, Nikita Khandelwal, Alex Meholic, Sahil Laheri, Akshay Sharma, Blake N. Johnson

**Affiliations:** 1 Department of Industrial and Systems Engineering, Virginia Tech, Blacksburg, Virginia, United States of America; 2 Department of Materials Science and Engineering, Virginia Tech, Blacksburg, Virginia, United States of America; 3 Macromolecules Innovation Institute, Virginia Tech, Blacksburg, Virginia, United States of America; 4 School of Neuroscience, Virginia Tech, Blacksburg, Virginia, United States of America; 5 School of Architecture + Design, Virginia Tech, Blacksburg, Virginia, United States of America; Istituto Italiano di Tecnologia Center for Micro BioRobotics, ITALY

## Abstract

Interfacing anatomically conformal electronic components, such as sensors, with biology is central to the creation of next-generation wearable systems for health care and human augmentation applications. Thus, there is a need to establish computer-aided design and manufacturing methods for producing personalized anatomically conformal systems, such as wearable devices and human-machine interfaces (HMIs). Here, we show that a three-dimensional (3D) scanning and 3D printing process enabled the design and fabrication of a sensor-integrated anatomical human-machine interface (AHMI) in the form of personalized prosthetic hands that contain anatomically conformal electrode arrays for children affected by amniotic band syndrome, a common birth defect. A methodology for identifying optimal scanning parameters was identified based on local and global metrics of registered point cloud data quality. This method identified an optimal rotational angle step size between adjacent 3D scans. The sensitivity of the optimization process to variations in organic shape (*i*.*e*., geometry) was examined by testing other anatomical structures, including a foot, an ear, and a porcine kidney. We found that personalization of the prosthetic interface increased the tissue-prosthesis contact area by 408% relative to the non-personalized devices. Conformal 3D printing of carbon nanotube-based polymer inks across the personalized AHMI facilitated the integration of electronic components, specifically, conformal sensor arrays for measuring the pressure distribution across the AHMI (*i*.*e*., the tissue-prosthesis interface). We found that the pressure across the AHMI exhibited a non-uniform distribution and became redistributed upon activation of the prosthetic hand’s grasping action. Overall, this work shows that the integration of 3D scanning and 3D printing processes offers the ability to design and fabricate wearable systems that contain sensor-integrated AHMIs.

## Introduction

Additive manufacturing, also called 3D printing, has emerged as a valuable fabrication process for creating personalized and anatomical biomedical devices by incorporating medical imaging data with computer-aided design (CAD) tools [1–7]. For example, 3D printed patient-specific anatomical tracheal implants have been developed for pediatric patients born with tracheobronchomalacia [8] 3D printed anatomical nerve regeneration pathways have also been used to regenerate mixed bifurcating peripheral nerve injuries in rats [4]. In addition to tissue regeneration applications, 3D printing has been used to fabricate patient-specific anatomical models for surgical testing applications [9–12].

Medical imaging data for 3D printing is often collected via magnetic resonance imaging (MRI) and computed tomography (CT) scanning [12–15]. 3D scanning techniques have also been used because of their relatively low cost, portability, flexibility in range and resolution, and user-friendliness [4, 16–19]. Thus, 3D scanners have been used across multiple industries, including healthcare and manufacturing, primarily for design and inspection applications [20–23]. 3D scanning techniques often differ regarding light sources, detectors, and sensing principles, but they are broadly categorized as laser- or patterned-based approaches [24–26]. Structured-light 3D scanning is a patterned-based approach for measuring the shape of an object based on the projection and reflection of light patterns [24, 26]. While laser 3D scanning has been used for medical imaging applications, structured-light 3D scanning offer advantages in speed, versatility, and price [26]. Structured-light 3D scanners have also facilitated micrometer- to millimeter-scale anatomical design of 3D printed anatomical devices [4, 17]. Thus, structured-light 3D scanning has the potential to become a transformative tool for designing anatomically conformal systems, such as prostheses, as it now enables personalization through anatomical digital models (*e*.*g*., of a patient’s limb structure) at lower cost and higher speed than MRI and CT scanning.

Over 1.6 million people are living with limb loss in the United States, and the number is expected to double by the year 2050 [27, 28]. Vascular diseases, such as diabetes, are currently the leading cause of limb loss and account for an estimated 54% of cases [27], while traumas resulting from events, such as car accidents and improvised explosive devices, account for an estimated 45% of cases [27]. Among 3D printing applications, the fabrication of prosthetic hands is an emerging area [29–31]. Prosthetic hands can be categorized as electric, myoelectric, and body-powered [29, 32]. For example, one study reviewed 58 3D printed upper limb prostheses and found that electric prostheses were superior in gripping tasks because of the capability to make a range of grasp types (*e*.*g*., power, precision, hook, spherical, tripod, and lateral grip) [29]. Several researchers also focused on improving prostheses finger movement by integrating servo motors [33], new tendon routing designs [34], and innovative kinematic designs of the thumb [34]. Bionic prosthetic hands derive function from the integration of electrical components with the user’s tissue, such as myoelectric control, and they often have bio-inspired geometric and mechanical designs [35]. Bionic hand reconstruction successfully restored hand function in three patients with global brachial plexus injury and lower root avulsions who had no alternative treatment [36]. However, bionic prostheses place a disproportionate economic burden on users, especially the families of children with amniotic band syndrome and similar birth defects, due to the initial cost and need to make size modifications throughout child development. For example, according to the non-profit organization Amputee Coalition, children generally need a new prosthesis every two years up to the age of 18 due to the growth of their bodies [37].

Among birth defects, amniotic band syndrome is especially common, occurring in approximately one of 1,000 births [38]. Amniotic band syndrome often results in limb malformation, commonly to the arm or hand [38]. Body-powered prosthetic hands have been frequently used for children with hand malformations caused by amniotic band syndrome or other congenital abnormalities because of their low cost, simplicity, maintainability relative to bionic prostheses, and large number of designs available for long transradial amputations [32]. While molding processes offer low-cost approaches for fabricating personalized tissue-prosthesis interfaces that could be potentially interfaced with non-personalized prostheses, 3D printing has emerged as a disruptive manufacturing process for creating low-cost prostheses for children with amniotic band syndrome [29, 39–42]. For example, online databases have been established to support the 3D printing of low-cost prostheses for children with birth defects, such as amniotic band syndrome (*e*.*g*., www.enablingthefuture.org). In parallel, prosthetic management for hand malformations remains an active area of research [43–45], in which the child’s age and fit of the prosthesis are often discussed as factors affecting prosthesis usage and cost. However, while 3D printing can be used for rapid prototyping of low-cost non-personalized prosthetic hands for children, amniotic band syndrome malformations are highly variable. Thus, approaches for personalizing generic digital models of prosthetic components could enable the fabrication of low-cost personalized prostheses for children with amniotic band syndrome.

In addition to fabricating anatomical biomedical devices [15], 3D printing enables continuous material deposition along non-planar tool paths, commonly referred to as conformal 3D printing, which is typically accomplished by printing in a support material or directly on an object [16, 35, 46–49]. While applications of conformal 3D printing are abundant and still emerging, conformal 3D printing has been used to create novel conformal and bionic devices [35, 50]. For example, microextrusion conformal 3D printing has been used to fabricate organ-conforming microfluidic devices for non-invasive isolation and profiling of biomarkers from whole organs [17] and stretchable tactile sensors [51]. Thus, conformal microextrusion 3D printing approaches could potentially enable the integration of electronic features, such as sensors, across anatomical human-machine interfaces (AHMIs), such as those found in personalized wearable systems.

Here, we describe an approach to create low-cost 3D printed personalized prostheses via an optimized 3D scanning and 3D printing method for applications to children with amniotic band syndrome. In addition to providing a methodology for optimizing 3D scanning parameter selection and design of the personalized prosthesis interface for multiple anatomical structures, conformal 3D printing was utilized to integrate conformal electrode arrays for measuring the pressure distribution across the tissue-prosthesis interface during use. 3D scanning and online CAD software were used to create a body-powered prosthetic hand that contained a personalized interface for a 12-year old child with a distal hand malformation caused by amniotic band syndrome. We found that personalization of the prosthesis geometry increased the tissue-prosthesis contact area. Ultimately, this work provides a new approach for the design and fabrication of low-cost 3D printed personalized prostheses with anatomically conformal electronic interfaces. The methods reported here can potentially be used to fabricate optimized personalized prostheses and wearable systems for a wide range of fundamental research and industrial applications.

## Materials and methods

### Materials

Alja-Safe^™^ and 300Q fast urethane resin were purchased from Smooth-On. Polymer filament (polylactic acid; PolyLite) was from Lulzbot. Assembly kits for the e-NABLE Raptor Hand were from 3D Universe. Multiwalled carbon nanotubes (CNTs) were from Cheaptubes.com. Polydimethylsiloxane (PDMS; Sylgard 184 Silicone Elastomer Kit) was from Dow Chemical. Copper tape was from 3M.

### Consent of human subjects

Participants for the study were recruited by flyers posted across the Virginia Tech campus. The 12-year old participant, recruited on February 2, 2017, was read a synopsis of the project’s objective and methods. Subsequently, the participant signed a child assent form. The child’s parents also signed an informed consent form that described the purpose of the study, procedures, risks, benefits, extent of anonymity and confidentiality, freedom to withdraw, approval of research, subject responsibilities, and subject’s permission. The individual in this manuscript has given written informed consent (as outlined in PLOS consent form) to publish these case details. All procedures were done in accordance with good practice as defined by the relevant national and local institutional healthcare bodies, and approved by the Virginia Tech Institutional Review Board (IRB).

### Reverse engineering of limb geometry via structured-light 3D scanning

Prior to 3D scanning, a cast of the participant’s hand was made using the Alja-Safe^™^-300Q resin system following the vendor-provided protocol. Subsequently, the polyurethane replica of the limb was scanned using a single camera, single projector structured-light 3D scanning system (SLS-2; HP). Prior to scanning, the system was calibrated using a 60 mm calibration grid following the vendor-provided protocol. The limb replica was scanned from a side-view with a stationary scanning system. A photograph of the experimental setup is provided in Figure A in S1 File. The limb replica was manually rotated after each scan by an angle *Δθ* using a turntable. The output from each 3D scanning measurement was a point cloud **P**, hereinafter referred to as a scan.

### Calculation of metrics for optimization of 3D scanning parameters

Two metrics were used to assess the quality of registered point cloud data, and thus, identify optimal 3D scanning parameters. *Scan Overlap Ratio (SOR) as a Local Quality Metric*: The limb replica was scanned from 0–360° using a constant rotational angle step size (*Δθ*). *Δθ* ranged from 5°—*θ*_*max*_, where *θ*_*max*_ was the maximum rotational angle at which the two scans could be successfully registered using the vendor-provided auto-alignment algorithm. Thus, this procedure resulted in a set of *n* scans (*i*.*e*., point clouds) **P**_i_ with surface area *A*_*i*_ for a given value of *Δθ*, where *n* = 360°/*Δθ*.

The overlap area between adjacent scans (*e*.*g*., a primary and secondary scan) **P**_1_ and **P**_i_ (*A*_*1i*_ = *A*_*1*_ ∩ *A*_*i*_) for a given value of *Δθ* was calculated using the following procedure, where *A*_*1*_ and *A*_*i*_ are the respective surface areas of the primary and secondary scans. The two scans were first registered using the vendor-provided software’s auto-alignment algorithm. The data in non-intersecting regions were then removed from the second scan using the trimming function of the software’s post-processing toolbox. The primary scan and the truncated second scan **P**_i_’ of surface area *A*_*i*_*’* were then exported to a commercially-available mesh editing software (Meshlab). The surface areas of the first scan (*A*_*1*_) and the trimmed second scan (*A*_*i*_*’* = *A*_*1i*_) were next calculated using a quality measure and computation filter for computing geometric measures within the software. This enabled calculation of SOR as *A*_*1i*_/*A*_*1*_. Iteration of this procedure for different values of *Δθ* then enabled construction of a plot of SOR vs. *Δθ*. Given the relationship of *SOR* vs. *Δθ* could depend on the initial projector-object orientation (*i*.*e*., the scanning perspective), we defined the object’s starting orientation as that which produced a primary scan of maximum surface area.

*Average Registration Error (ARE) as a Global Quality Metric*: The effect of *Δθ* on the dimensional accuracy of registered 3D models was analyzed in terms of the ARE among a globally assembled set of scans **P**_i_ acquired at each *Δθ*. All scans **P**_i_ for a given value of *Δθ* were first registered using an iterative closest point (ICP) algorithm for pairwise local alignment followed by global alignment using a global minimization algorithm that distributed the residual error among all pairs using Meshlab [52–55]. The effect of *Δθ* on the dimensional accuracy of the reconstructed 3D model was then analyzed in terms of the ARE, calculated as the average residual error after the global minimization process (*i*.*e*., a global alignment) [52, 54, 55]. The ARE was normalized to facilitate comparison among different replicas based on the maximum value obtained over the ranges of *Δθ* that led to successful global alignment of point cloud data. The aforementioned procedure was repeated using replicas (i.e., molds) of an adult human ear (female), adult human hand (male), adult human foot (male), and adult porcine kidney (female) to examine the dependence of the scanning parameter selection across anatomical structures of varying organic shape. The molds for the ear, hand, and foot were obtained using the aforementioned molding procedure. The mold of the kidney was obtained using previously reported methods [17]. The relationship between SOR and ARE, the respective local and global quality metrics, were then used to identify optimal scanning parameters, here, the optimal rotational angle step size (*Δθ*_*opt*_) for producing dimensionally accurate 3D models based on the minimum amount of required point cloud data.

### Computer-aided design of the 3D printable personalized prosthetic hand

The digital models for the components associated with a non-personalized prosthetic hand for amniotic band syndrome defects (Raptor Hand; e-NABLE) were first downloaded from an online database (www.thingiverse.com). This assembly contained 31 different parts, including wrist, palm, and finger components (STL formats). Online CAD software (TinkerCAD) was then used to design the digital models needed for the personalized prosthetic hand, given its flexibility for manipulating STL files and wide accessibility. Prior to modification, all components were scaled by a scale factor (150%) that was calculated based on the ratio of the width of the participant’s wrist to the width of the opening of the non-personalized wrist component. The downloaded palm component was then opened in the CAD software. The palm component was the focus of the CAD process as it was the only component that interfaced with the distal end of the participant’s hand (the location of the amniotic band syndrome malformation). A rectangular box that was large enough to enclose the interior palm cavity of the non-personalized palm component was then created in the CAD software’s graphic user interface (GUI) using the ‘solid box’ command. The box was then modified to fit in the original palm cavity by subtracting the two domains as follows. First, the box was translated until it filled the palm cavity and overlapped with cavity boundaries. The box was then trimmed via a subtractive process using the software’s ‘hole’ command with the original palm component serving as the hole. Subsequently, the original palm domain was deleted from the GUI. This process resulted in a form-fitting palm insert component that could be modified with the anatomical data collected via the 3D scanning process. The digital model of the participant’s hand created by the aforementioned 3D scanning process was then opened in the same GUI as the previously created palm insert component. Similar to the process for creating the form-fitting palm insert, the hand model and the form-fitting palm insert component were used to create a personalized palm insert by subtracting the two domains as follows. First, the hand model was translated (*i*.*e*., shifted) until it partially overlapped with the form-fitting palm insert component on the opposite side that interfaces with the original palm component (*i*.*e*., the prosthesis). The form-fitting palm insert was then trimmed via a subtractive process using the software’s ‘hole’ command with the hand model serving as the hole. Subsequently, the hand model was deleted from the GUI. This process resulted in a personalized palm insert. The personalized palm insert and the original palm component were then opened in the GUI. Subsequently, the two domains were merged using the software’s “group” command, resulting in a personalized palm component. The model was then saved in STL format for 3D printing.

### 3D printing and assembly of prosthetic hands

The components for both a non-personalized (all original components) and a personalized prosthetic hand (all original components except with the non-personalized palm component replaced with the personalized palm component) were printed using multiple commercially-available polymer extrusion 3D printers (either a Prusa i3 MK2 from Prusa Research or a Mini 2 from Lulzbot). Parts were printed with a 0.18 mm layer height, 10% infill density (for support structures), and speed of 30 mm/s for wall printing and 40 mm/s for infill printing. Following printing, the support structures were manually removed. The components were then assembled using the commercially-available assembly kits following online instructions (www.handchallenge.com and www.thingiverse.com).

### Dimensional comparison of 3D scanning data and limb geometry

The accuracy of the 3D scanning process was assessed by comparing the dimensions of the digital model generated from 3D scanning with the dimensions of the participant’s limb (represented by the hand cast). The width of each of the five nubs and palm were quantified at their widest points to provide metrics for analysis of the dimensional accuracy of the 3D scanning process. The 3D scanning data of the hand used for creating the personalized palm component was analyzed using an image processing software (ImageJ; National Institutes of Health) using a line measurement command. A caliper was used to measure the same metrics from the mold of the participant’s hand.

### Assessment of tissue-prosthesis contact area

The effect of personalization on the tissue-prosthesis contact area was calculated by assembly modeling with Rhinoceros (Rhino 6), which is an approach for positioning the components using absolute coordinate placement or relative position. The imported CAD models included the personalized palm component, the non-personalized palm component, and the 3D model of the participant’s hand that was constructed via 3D scanning. The relative position of the prosthesis component with respect to the limb was set based on the orientation observed via photography during use by the participant. The tissue-prosthesis contact area (*A*_*contact*_) was defined as the intersection area between the digital model of the participant’s limb and each of the two palm components of the prosthetic hand.

### Characterization of PDMS-carbon nanotube inks as pressure sensors

Polymer nanocomposite inks were prepared over the concentration range of 1–20 wt% in 10:1 base: agent ratio PDMS. The inks were mixed in a centrifugal mixer (ARE-310; Thinky). For testing, a 1 mm thick film of ink (*w*) was 3D printed onto glass slides using the parameters described for conformal electrode printing (we note that the 20 wt% ink was too viscous for extrusion using a digital pressure regulator and thus, was hand printed). Samples were fabricated using inks ofvarying CNT filler content. Following 3D printing, the samples were cured. The resistivity of the PDMS-CNT inks was then measured using a four-point probe method (SP4-40085TRJ; Signatone) and a power source meter (2450 SourceMeter; Keithley) at 1 A. The body resistivity associated with a sample of finite thickness *w* was calculated based on an infinite slice assumption using the relation (*V*/*I*)*w*[π/ln(2)]*F*(*w*/*s*), where *V*/*I* is the resistance (here, measured by the source meter), *w* is the sample thickness, *s* is the four point probe spacing (here, *s* = 1.33 mm), and *F*(*w*/*s*) is a correction factor that approaches unity as *w* approaches zero [56]. The pressure sensitivity of the 3D printed polymer electrode arrays was characterized by measuring the resistance of a single pair of electrode terminals under a range of applied forces, given the contact area remained constant throughout the measurement. For testing, a pair of polymer electrodes was 3D printed onto a glass substrate using the same parameters that were used for conformal 3D printing. Electrical contact between the electrode junctions was created by first placing copper tape across the electrode terminals. Subsequently, the resistance of the two-electrode circuit was measured using a multimeter (Fluke, 289 True RMS Multimeter) as the applied pressure across the electrode junction was varied by placing calibrated weights from a calibration set (Neewer 205) on top of the electrodes. The applied force was calculated as the product of the mass of the weight and the acceleration due to gravity. Details on electrode area are provided in the following section.

### Conformal 3D printing of anatomical electrode arrays

Conformal electrode arrays were 3D printed using a PDMS-CNT composite ink in 10:1 base: agent ratio PDMS. A CNT filler content of 15 wt% was used for printing. A 2D tool path for the conformal electrode arrays was first designed in a commercially-available CAD/computer-aided manufacturing (CAM) software (Rhino 6; Rhinoceros). The conformal electrode array contained one pressure sensor per metacarpal in the hand. Each pressure sensor consisted of a pair of 4 mm diameter pad electrodes (center-to-center distance = 4 mm) and two associated conductive leads of different length (26 and 15 mm). Thus, the anatomically conformal electrode array contained a total of five pressure sensors and 10 electrodes. Subsequently, the 3D tool path associated with the conformal electrode array was obtained using the associated 2D tool path of the array and the digital model of the personalized palm component using Rhino 6. Before printing of the conformal electrode arrays, the inner surface of the personalized palm component was coated using a thin layer of PDMS (10:1 base: agent ratio). PDMS was applied using a paintbrush to both smooth the surface for subsequent conformal printing and promote adhesion of the PDMS-CNT ink. The ink was loaded into 3cc syringe printing barrels with a 16-gauge tapered tip to fabricate the conformal electrode arrays. The electrode arrays were then printed using a custom low-cost microextrusion 3D printing system created by mounting the microextrusion printing barrel to the heated extruder system already present in the low-cost plastic 3D printing systems that were used for prosthesis 3D printing. The 3D printer from Lulzbot was used in all conformal 3D printing studies. Deposition of the CNT-PDMS ink during printing was accomplished using a digital pressure regulator (DC100; Fisnar). The conformal electrodes were printed at a speed of 3.3 mm/s using a pressure of 2 psi. The personalized palm component was then heated overnight at 90 °C to cure the ink. Following curing, the diameter of the 3D printed conformal filaments was measured based on photographs of the 3D printed conformal electrode arrays using the feature measurement tool in a commercially-available image processing software (ImageJ; NIH).

### Measurement of the pressure distribution across the personalized prosthesis interface

Prior to placing their hand in the personalized prosthesis, the dorsal side of the participant’s hand was wrapped in a flexible thin film of plastic covered with copper tape to eliminate potential effects of skin moisture on the sensor signal. Subsequently, the straps on the prosthetic hand were adjusted to fit the participant. The participant was then asked to place the prosthetic hand on a rigid flat table with the palmar side facing upward and the wrist relaxed in a straight position, referred to as the ‘relaxed’ position. The response of each sensor (1–5) was then measured by recording the resistance across each electrode pair. The signal reported for each electrode is the average of *n* = 4 measurements recorded over a one-minute interval. The participant was then asked to flex their wrist, which created a grasping and flexing action in the prosthetic hand, while the response from each pressure sensor in the array was recorded using the aforementioned procedure. This was referred to as the ‘flexed’ position. During this procedure, the fingers of the prosthetic hand were scanned from a side perspective to verify that personalization did not impede the body-powered grasping action.

## Results and discussion

### Description of the participant’s limb anatomy

**Fig 1:**
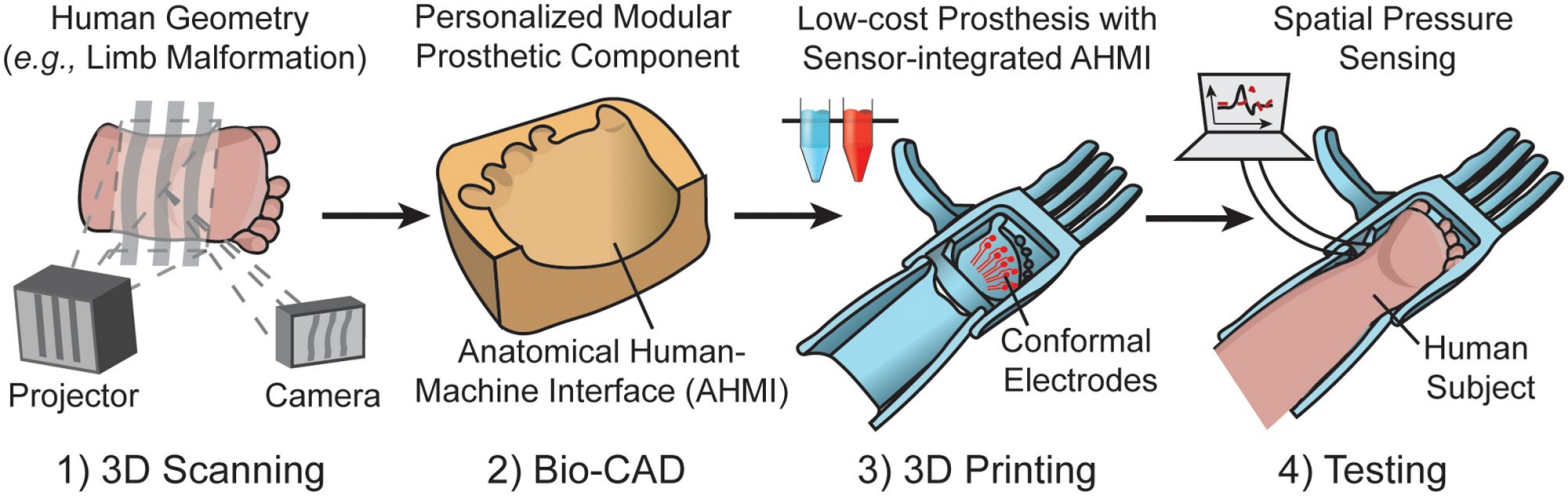
3D scanning and 3D printing create a flexible computer-aided manufacturing platform for design and rapid prototyping of personalized wearable systems with a focus on the creation of low-cost personalized prosthetic hands with sensor-integrated anatomical human-machine interfaces (AHMIs) for children with amniotic band syndrome.

**Fig 2:**
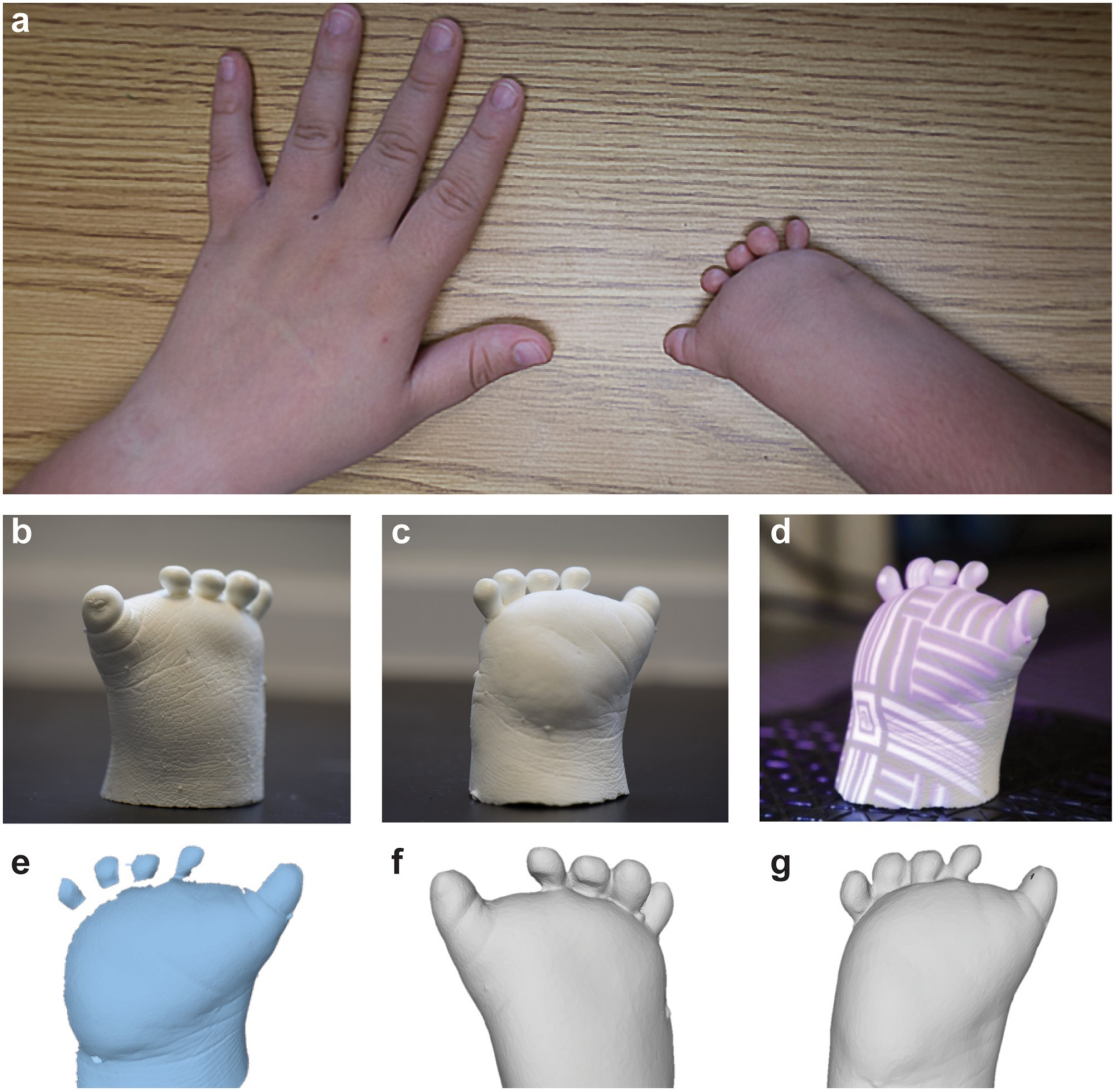
Reverse engineering of the limb malformation via structured-light scanning. a) Photograph of the participant’s limb malformation associated with amniotic band syndrome. Photographs of the hand cast from dorsal (b) and palmar perspectives (c). d) Photograph of the hand cast during 3D scanning showing the interaction between a representative structured-light pattern and the object. e) Point cloud data from a single scan highlighting the 3D scanning process. Assembled digital models of the participant’s hand geometry shown from dorsal (f) and palmar (g) perspectives.

Amniotic band syndrome results from limb entanglement with amniotic fibers *in utero*. This condition typically results in a malformation of limbs, such as hand and feet. As shown in Fig 1, the premise of this work is that 3D scanning and 3D printing can facilitate the design and fabrication of low-cost personalized prosthetic hands with anatomically conformal electronic interfaces for children with amniotic band syndrome. As shown in Fig 2A, the participant’s right hand was affected by amniotic band syndrome. Five ‘nubs’ were visible at the distal end of the hand. This type of malformation is common among children born with amniotic band syndrome and other types of congenital malformations, such as symbrachydactyly, and motivates the design of currently available low-cost 3D printed prosthetic hands (*e*.*g*., available through prostheses databases such as e-NABLE). Having characterized the participant’s limb anatomy and identified the corresponding available non-personalized prosthesis model using the online databases, we next examined the ability to reconstruct a digital template of the participant’s limb using 3D scanning for use in personalizing the generic digital models.

### Reverse engineering of limb geometry via 3D scanning

To date, structured-light 3D scanning has emerged as a complementary technique to 3D printing that has enabled device personalization and anatomical matching [17]. Therefore, structured-light 3D scanning was used to generate a digital model of the participant’s limb for use in designing a personalized prosthetic hand. As shown in Fig 2B and 2C, the first step of the process involved making a cast of the participant’s hand. Structured-light 3D scanning processes work through a reflection principle by which a structured-light pattern is projected onto a non-transparent or -reflective object and viewed through a camera (see Fig 2D). In order to highlight the 3D scanning process, the point cloud data from a single scan is shown in Fig 2E. The data missing between the nubs and palm were reconstructed by registration of additional scans taken from different perspectives.

Performing the 3D scanning process using a rotational angle step size (*Δθ*) that provided substantial overlap area between adjacent scans, here 30°, produced a fully assembled model shown from the dorsal and palmar perspectives in Fig 2F and 2G, respectively. The data in Fig 2E–2G also show the presence of five ‘nubs’ at the distal end of the hand. This process demonstrated the effectiveness of 3D scanning for creating digital templates of limb geometry, while also highlighting the anatomical geometry and features that would ultimately become interfaced with a target wearable system or machine. We note that while direct 3D scanning of the tissue to obtain the limb geometry would simplify the approach, we found this to be difficult due to movement of the child’s limb during scanning. Further work is needed to examine direct limb scanning approaches that can account for limb movements during data acquisition. A comparison of the dimensions of the scan data with the hand cast suggested that the dimensions of the scanning data were accurate within 96% of the limb dimensions.

### Identification of optimal scanning parameters and testing with various anatomical structures

**Fig 3:**
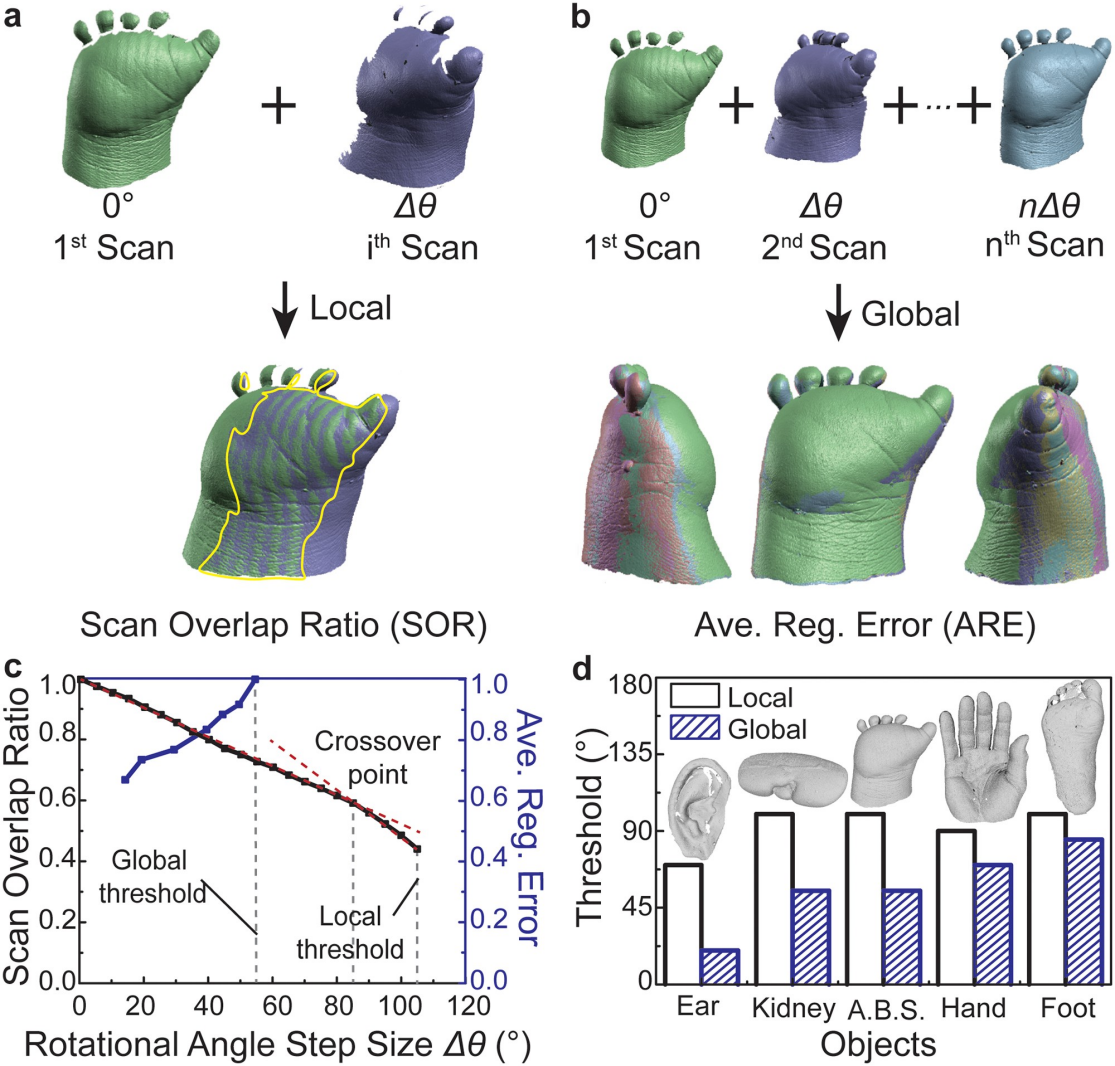
Methodology for selection of optimal scanning parameters based on scan quality assessment metrics and sensitivity to multiple anatomical structures. a) Description of the scan overlap ratio (SOR) between two registered scans as a local metric of scan quality. b) Description of average registration error (ARE) among a set of globally registered scans as a global metric of scan quality. c) The relationship between scan quality metrics and the scanning parameter of interest (here, the rotational angle step size (*Δθ*)). d) Sensitivity analysis of local and global alignment thresholds to variations in anatomical structure tested using five different anatomical structures (abbreviation: amniotic band syndrome (A.B.S.)).

Having demonstrated the principle of using 3D scanning to reverse engineer 3D digital models of limb geometry, we next examined a procedure for identifying the optimal scanning parameters. While there could be multiple approaches for optimizing scanning parameters, here we focused on identifying scanning parameters, specifically *Δθ*, that produce dimensionally accurate 3D models based on the minimum amount of required point cloud data. This is an important consideration as the value of *Δθ* affects the amount of point cloud data generated. We remind the reader that the number of scans requiring global alignment is based directly on the value of *Δθ* as *n* = 360°/ *Δθ*. As shown in Fig 3A and 3B, model assembly from point cloud data is based on the principle of collecting successive scans that provide sufficient similarities in structure for the scans to be registered (*i*.*e*., aligned). However, while many values of *Δθ* can create produce overlapping primary and secondary scans, we examined whether it was possible to identify an optimal value of *Δθ* based on the objective of minimizing the amount of data needed to assemble a dimensionally accurate 3D model. Specifically, it is of interest to determine the maximum rotational angle step size (*Δθ*_*opt*_) between successive scans as this would minimize the total number of collected scans (*n*), and thus, the required computing power and the post-processing time. We note that this is an important consideration as previous studies using 3D scanning in 3D printing applications typically report the rotational angle step size used for reverse engineering [17, 57, 58], but do not discuss optimization of scanning parameter selection. Thus, we next examined an approach for identifying *Δθ*_*opt*_ for reverse engineering 3D models associated with hand malformations caused by amniotic band syndrome.

As shown in Fig 3C, the scan overlap ratio (SOR) decreased from 1.0 to 0.45 over the range of *Δθ* = 0 to 105°. We found that a step size greater than 105° did not facilitate scan registration, which provided a threshold value for *Δθ*. Thus, this was referred to as a ‘local’ threshold because it did not involve data acquired from the object’s entire form, but rather two scans of the object separated by a rotational step *Δθ* that represent its local geometry. The data exhibited two linear regions characterized by slopes of different magnitude. As shown in Fig 3C, the crossover point between the two linear regions occurred at *Δθ* = 85°. Given changes in *Δθ* caused relatively larger changes in SOR above the crossover point (*i*.*e*., the slope of SOR vs. *Δθ* exhibited a larger absolute value above the crossover point), this suggested that the location of the crossover point could serve as *Δθ*_*opt*_ in applications when assembly of a complete 3D model is not required.

For applications requiring assembly of a complete 3D model, we examined the dependence of the average registration error (ARE) on *Δθ*. While SOR provided insight into scan quality based on an object’s partial geometry, ARE provided a measure of the alignment quality among a set of globally assembled scans, which influences the model’s dimensional accuracy. As shown in Fig 3B, the number of scans (*n*) in a complete set that were registered to generate a 3D model was dependent on *Δθ*. As shown in Fig 3C, ARE increased relatively linearly over the range of *Δθ* = 15–55°. The study also showed that step sizes greater than *Δθ* = 55° did not facilitate convergence of the registration algorithm, which provided a second threshold for *Δθ*, referred to as the global threshold in Fig 3C. Thus, given the global threshold occurred within the region of local stability, this suggested that the location could serve as *Δθ*_*opt*_, specifically the maximum step size in rotational angle.

Given the previous methodology could potentially be applied to a number of anatomical structures in future applications, we next examined the trends of the local and global thresholds across multiple anatomical structures. We selected different structures to represent a range of geometric shape factors, length scales, and feature sizes, including an adult foot, hand, and ear as well as an adult porcine kidney. As shown in Fig 3D, the local and global thresholds varied considerably across the set of objects. The anatomical structures with the smallest features, such as the ear, exhibited the lowest relative values of local and global thresholds at 70 and 20°, respectively. Detailed renderings of the scanned objects and their corresponding molds are presented in Figure B in S1 File. We also found that the global thresholds among structures with highly dissimilar geometry (*e*.*g*., the ear and the foot) differed substantially. For example, as the global thresholds for the ear and foot were 20 and 90°, respectively. This result illustrated a methodology for identifying optimal scanning parameters, since it suggested that the optimal scanning parameters were dependent on the object’s size and geometry.

### Computer-aided design of a personalized prosthetic hand using 3D scanning data

**Fig 4:**
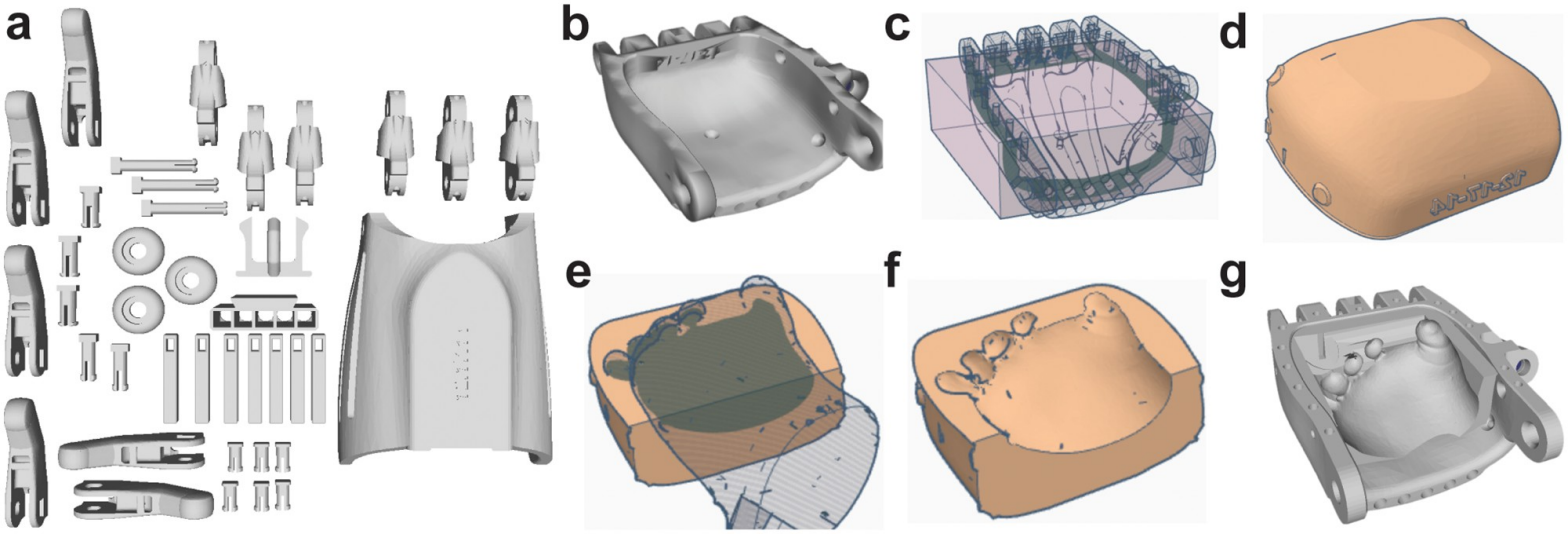
Computer-aided design process for the personalized prosthetic hand. a) Components of a right-hand prosthetic hand prior to personalization via the 3D scanning-CAD process. b) Highlight of the only component that interfaces directly with the tissue affected by the amniotic band syndrome malformation prior to personalization (referred to as the original palm component). Use of a subtractive CAD operation (c) to create a form-fitting palm insert to the original palm component (d). Use of a subtractive CAD operation (e) to create a personalized palm insert (f). g) Highlight of the personalized palm component formed by interfacing the personalized palm insert shown in panel (f) with the original palm component shown in panel (b).

Having reconstructed a digital template of the participant’s limb malformation via 3D scanning using optimal scanning parameters, we next used a widely accessible online CAD software to design a personalized 3D printable prosthetic hand based on the non-personalized prosthetic hand models that were available through the online prostheses database e-NABLE. As shown schematically in Fig 4A, the prosthesis assembly for children with distal hand amniotic band syndrome malformations contained 31 components. As shown by inspection of the participant’s limb malformation and the 31 components that comprised the prosthetic hand model, only one component contacted the limb in a region that was affected by amniotic band syndrome, specifically the original palm component (see Fig 4B). Thus, a series of CAD operations (see Fig 4C–4F) were used to create a personalized palm insert (see Fig 4F). The original palm component was then modified with this personalized palm insert to yield a personalized palm component (see Fig 4G). Thus, the set of components shown in Fig 4A, with the personalized palm component used in place of the original palm component, were then used to 3D print and assemble a personalized prosthetic hand.

### 3D printing of personalized prosthetic hands

**Fig 5:**
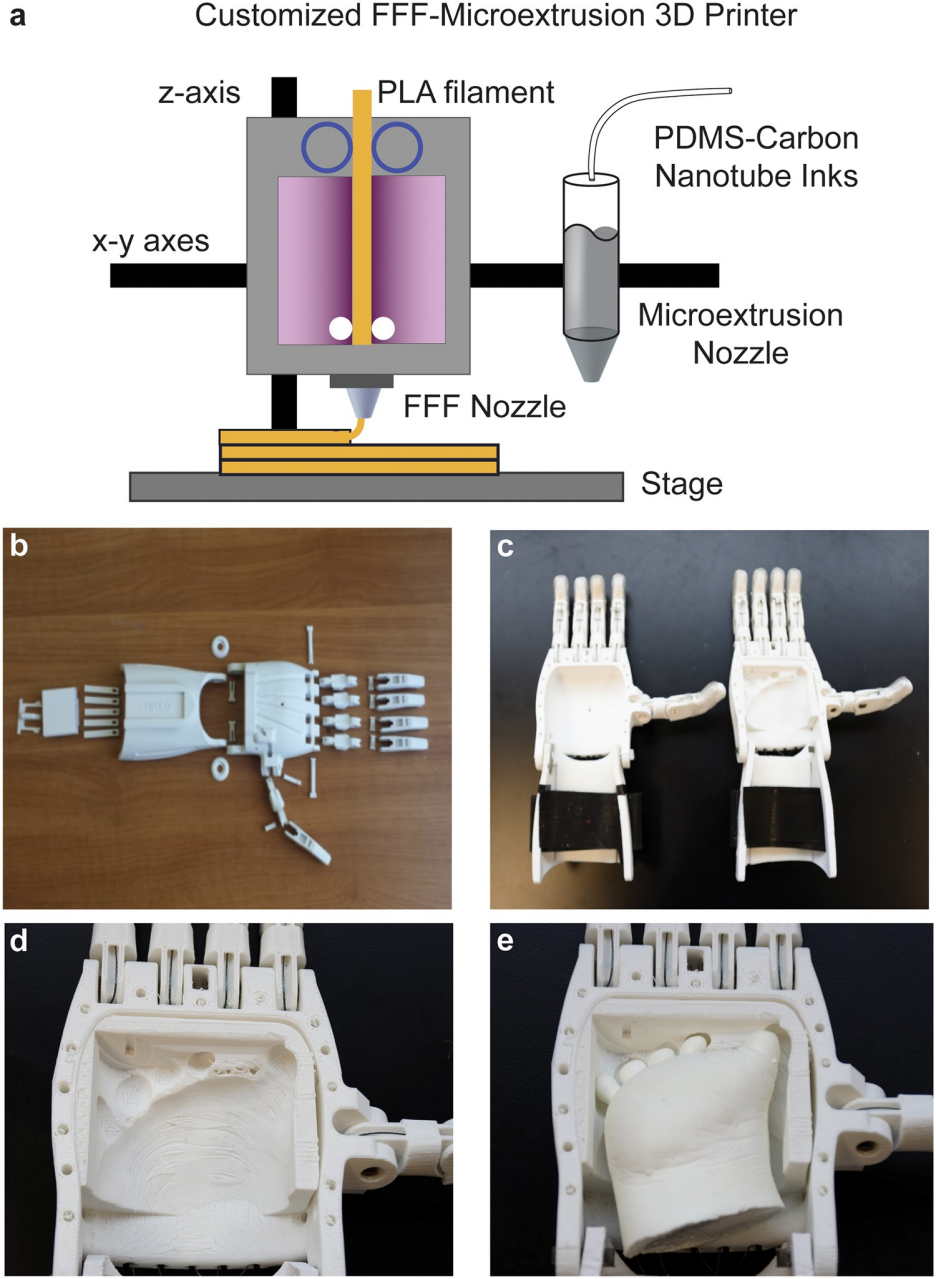
Fabrication of the 3D printed personalized prosthetic hand. a) Schematic of a custom low-cost FFF-microextrusion printer used for fabrication of the 3D printed personalized prosthetic hands and conformal electrode array 3D printing. b) Photograph of all the printed prosthetic hand components prior to assembly. c) Photograph of the assembled non-personalized prosthetic hand (left) next to the personalized prosthetic hand (right). Close-up views of the personalized interface in the absence (d) and presence (e) of the hand cast.

With the continued development of 3D printing technology and materials, 3D printers are becoming increasingly affordable, with printers capable of fabricating prosthetic hands available in the range of $300 to $1,500. Polymer extrusion 3D printers are among the most inexpensive systems. As shown schematically in Fig 5A, polymer extrusion 3D printers continuously extrude preprocessed thermoplastic filaments through a heated nozzle leading to layer-by-layer deposition of the polymer in a tool path that was generated from a corresponding digital model of the 3D printed part. Importantly, as shown schematically in Fig 5A, the polymer extrusion 3D printer exhibited sufficient build volumes that enabled mounting of additional tools, specifically, a syringe for conformal microextrusion 3D printing of anatomically conformal electronics. The individual components of the prosthetic hand fabricated using a polymer extrusion 3D printer are shown in Fig 5B. Previous research has shown that most patients indicate the aesthetic appearance of an amputated finger plays a more important role than function [59]. Thus, the ability to rapidly prototype bio-inspired prosthetic hands of various mechanical design and material color makes 3D printing a promising approach for fabrication of low-cost prostheses for children. Having 3D printed the components of the non-personalized and personalized prosthetic hands, we next assembled the hands for use by the participant as shown in Fig 5C–5E. We note that the prosthetic hand’s grasping action is actuated by the user’s wrist flexion through a combination of elastic and tensioning cables. As shown in Fig 5D–5E, the anatomical geometry of the participant’s limb was preserved in the 3D printed personalized palm component.

### Assessing the effect of personalization on tissue-prosthesis contact area

**Fig 6:**
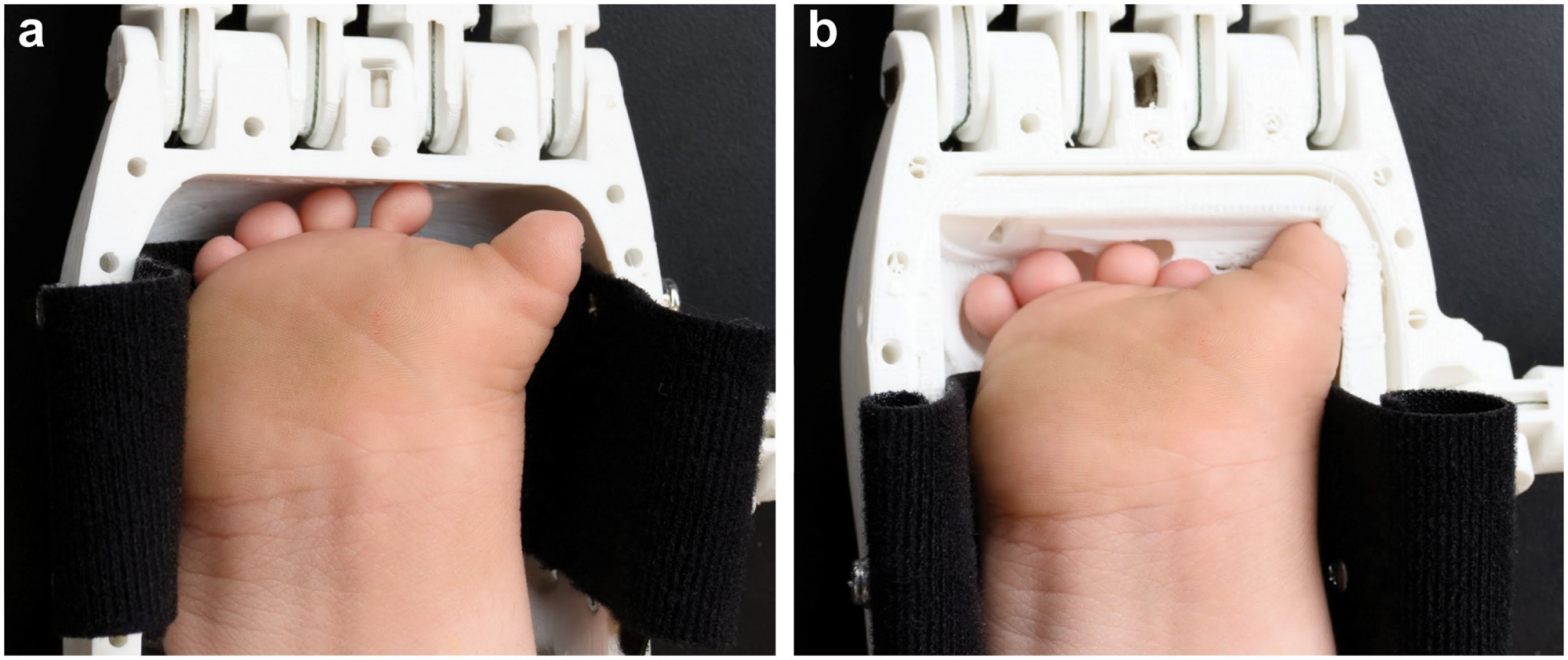
Verification of the 3D printed personalized prosthetic hand’s function via 3D scanning. Photographs of the participant’s hand inserted into both the non-personalized (a) and personalized prostheses (b).

As shown by comparison of Fig 6A and 6B, the personalized prosthetic hand exhibited an improved fit to the participant’s hand relative to the non-personalized design based on qualitative assessment via photography. For example, the form-fitting nature of the personalized prosthetic hand was visible toward the distal end of the participant’s hand in Fig 6B. Having exposed the participant to both prosthesis designs, we next verified that personalization of the palm insert component did not impede its body-powered grasping action. The participant was able to actuate the body-powered grasping action through wrist flexion. Confirmation of the grasping action was also verified using 3D scanning (see Figure C in S1 File).

**Fig 7:**
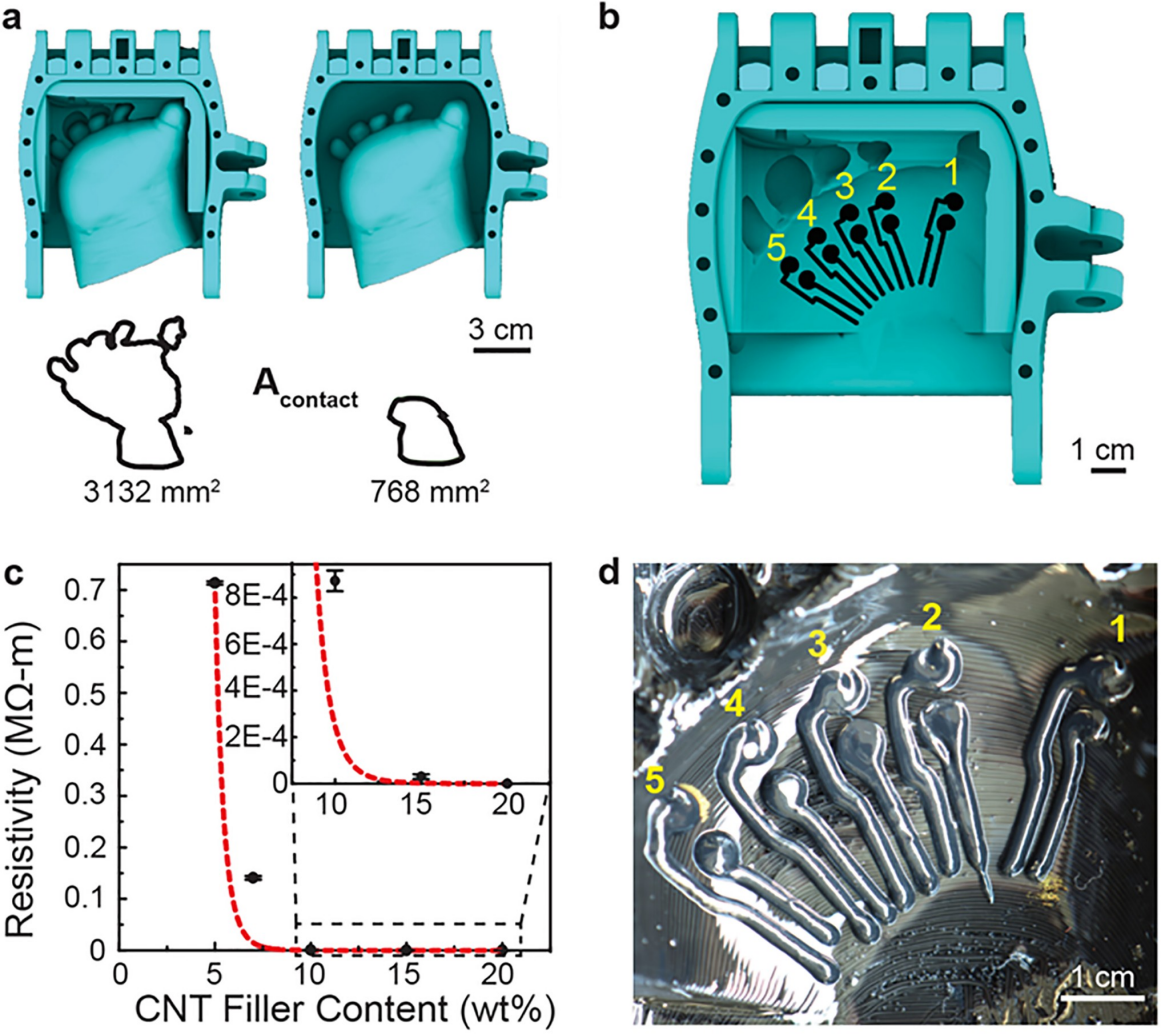
Integration of anatomically conformal electrode arrays into the personalized tissue interface via conformal 3D printing. a) Results of the CAD-based analysis showing the effect of personalization on the tissue-prosthesis contact area (*A*_*contact*_*)*. b) Schematic showing the 2D tool path of the 3D printed conformal electrode arrays for interface with each metacarpal on the dorsal side of the hand. c) Experimental data showing the effect of CNT filler content on resistivity of the PDMS-CNT ink, including a line of best-fit (dashed red line). d) Photograph of the 3D printed conformal electrode array corresponding to the tool path shown in panel (b).

Having verified that personalization of the palm insert did not impede the prosthetic hand’s grasping mechanism, we next examined the effect of personalization on the tissue-prosthesis contact area. The tissue-prosthesis contact area provides a useful parameter to be considered in the analysis of fit, and thus, is likely to affect comfort and function. For example, increasing the tissue-prosthesis contact would reduce the pressure exerted on the user’s limb by increasing the surface area over which loading is distributed. Furthermore, increasing the tissue-prosthesis contact area would increase the area available for electronic interface with the user’s limb. As shown in Fig 7A, we found that personalization increased the tissue-prosthesis contact area (*A*_*contact*_) by 408% relative to the non-personalized design. Specifically, the tissue-prosthesis contact areas of the non-personalized and personalized designs were 768 and 3,132 mm^2^, respectively. This result suggests that personalization could reduce the pressure on a user’s limb relative to the pressure found in a non-personalized prosthesis.

### 3D printing of anatomically conformal electrode arrays and sensor characterization

In addition to potentially reducing the pressure exerted on the user’s limb, increasing the tissue-prosthesis contact area has implications regarding potential improvement to prosthesis comfort and function. For example, increasing the tissue-prosthesis contact area creates new opportunities for integrating components required in bionic systems, such as sensors. The ability to understand the pressure distribution across the AHMI via integrated sensors would provide useful information for understanding the biomechanics associated with personalized wearable systems as well as improving their comfort and function. To illustrate the potential for integrating sensors into the prosthetic hand in a low-cost fabrication format for potential pressure mapping applications, we next designed a conformal electrode array to interface with the dorsal side of the user’s hand. As shown schematically in Fig 7B, the five pairs of conformal electrodes extended longitudinally along the hand’s metacarpals.

We next examined the effect of CNT filler content on the resistivity of cured PDMS-CNT inks based on their previous use in strain and pressure sensing applications [60–62]. The data in Fig 7C show the effect of CNT filler content on the resistivity of the PDMS-CNT ink. We found that the resistivity of the PDMS-CNT ink decreased with increasing CNT content. As shown in Fig 7C, the resistivity exhibited a sharp change at a CNT content of approximately 10%. Beyond this range, the resistivity remained relatively constant at values below 1 kΩ-m. Such values compare reasonably with those obtained from CNT filler contents of 7–8% used in previous PDMS-CNT-based pressure sensing studies [60]. In that study, they found the devices exhibited a pressure sensitivity of 500 Pa, which was found to be smaller than the pressure associated with a small touch to the human skin (10 kPa) [60, 63, 64]. The applied forces used for testing ranged from 0–1.4 N in steps of 350 mN, with equivalent pressures ranging from 0 to 9 kPa [60], which compare reasonably with the other applications in biomonitoring and electronic skin that require high sensitivity in the low-pressure regime <10 kPa [65]. Thus, we examined the 3D printed sensors’ response over a similar range of applied forces.

While a CNT filler content of 10 wt% could have provided a useful sensor for mapping pressure distributions across the personalized interfaces of prosthetic hands based on previous research, the resulting PDMS-CMT ink did not exhibit the rheological properties needed to facilitate conformal 3D printing of high-quality electrodes. Given conformal 3D printing involves deposition of inks on non-flat surfaces (*i*.*e*., non-planar 3D printing), material can potentially flow down the surface through a falling film effect (*i*.*e*., flow under a gravitational load), resulting in poor quality of the conformally 3D printed material. For example, spreading effects due to material flow on curved substrates have been previously discussed in research on 3D printed conformal antennas [66]. Inks that exhibit Hershel-Bulkley type rheological properties are widely accepted as ideal candidates for 3D printing, especially for conformal printing applications. For example, they enable the 3D printing of free-standing macroscopic structures on flat and non-flat surfaces whose final form after curing exhibits a high dimensional accuracy with the originating path code (*i*.*e*., digital model). In our previous work, we found that Room-Temperature-Vulcanizing (RTV) silicone exhibited a yield stress that was sufficient to enable conformal 3D printing of form-fitting anatomical microfluidic devices for non-invasive isolation and profiling of biomarkers from organs [17]. Thus, it was of interest to determine the concentration at which the PDMS-CNT system exhibited a sharp change in viscosity or yield stress, as such would identify an optimal CNT filler content for conformal 3D printing. We found that a CNT filler content of 15 wt% resulted in inks that exhibited yield stresses capable of preventing the flow of ink after deposition. Thus, given a filler content of 15 wt% was also above the conductivity threshold identified in Fig 7C, this concentration was selected for 3D printing of the conformal electrode arrays as it provided sufficient yield stress for 3D printing and conductivity for pressure sensing based on previous work. One previous study found that PDMS-CNT composites exhibited a sharp increase in viscosity at filler contents ranging from 3–4 wt% [67]. We note that the wide range could be due to differences in: 1) the type and source of CNTs; 2) the concentration of the PDMS (*i*.*e*., base-hardener ratio); and 3) the processing method utilized for preparation (*e*.*g*., mixing techniques) [67]. Fig 7D shows a photograph of the conformally 3D printed PDMS-CNT electrode array containing five pressure sensors in the personalized 3D printed prosthetic hand.

### Spatially-resolved pressure sensing at the personalized prosthesis-tissue interface using conformally printed sensor arrays

**Fig 8:**
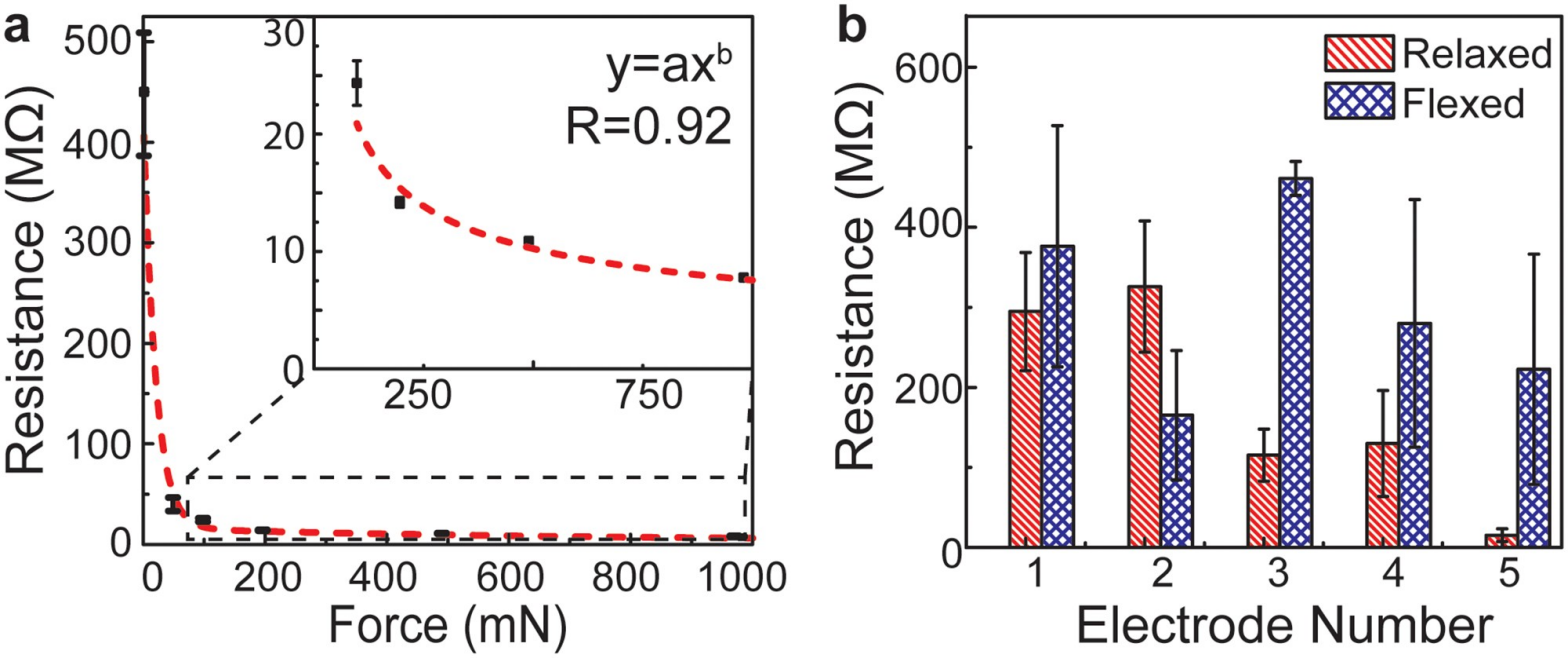
Sensor characterization and mapping of pressure distributions across the personalized prosthesis-tissue interface using the anatomically conformal sensor array. a) Validation of the 3D printed sensor’s dynamic range. b) Response of the anatomically conformal 3D printed sensor array to body-powered actuation of the prosthetic hand’s grasping action by the participant shows a non-uniform pressure distribution that became re-distributed upon actuation.

As shown in Fig 8A, the 3D printed electrodes functioned as pressure sensors over the range of 0 to 980 mN based on previous work [60]. The sensor’s response above 980 mN was not investigated in this study. As shown in Fig 8A, the resistance across the electrode terminals changed from 447 ± 61 to 7.8 ± 0.1 MΩ over the range of applied forces. A power-law model fit the data with a high confidence level (R^2^ = 0.92). Having validated the anatomically conformal electrode’s ability to function as a 3D printed pressure sensor in a controlled setting, we next examined the response of the electrode array integrated within the 3D printed personalized prosthetic hand to better understand the pressure distribution that occurred across user’s limb while wearing the prosthesis and during use.

A photograph of the participant wearing the 3D printed personalized ‘bionic’ prosthesis during testing (*i*.*e*., the prosthesis with the anatomically conformal electrode arrays) is provided in Figure D in S1 File. As shown in Fig 8B, the resistances, and thus, forces and pressures, measured across each electrode were significantly different depending on whether the hand was in a ‘relaxed’ or ‘flexed’ position. For example, the resistance measured across each electrode pair while the participant’s wrist was relaxed (*i*.*e*., straight) ranged from 326 ± 82 to 15.3 ± 8.0 MΩ. Considering the relationship between sensor resistance and corresponding applied force shown in Fig 8, the minimum and maximum forces, and thus, pressures, were measured on electrodes 2 and 5, respectively. This showed that while the geometric fit between the prosthetic hand and the user’s limb exhibited anatomical matching, the pressure distribution was non-uniform across the interface (here across the hand’s dorsal side). As shown in Fig 8B, a non-uniform distribution became redistributed when the participant engaged the prosthetic hand into the flexed position. For example, the resistances measured exhibited a similar range of 461 ± 21 to 165 ± 81 MΩ, but the minimum and maximum pressures were found in different locations relative to the relaxed position (electrodes 3 and 2, respectively). This result suggests that while the pressure distribution remained non-uniform across the participant’s limb while the prosthetic hand was in both the relaxed and flexed positions, the forces were redistributed during the transition between the two positions. We remind the reader that these conclusions assume that any potential effects of curvature on pressure sensitivity among different sensors are negligible. To further substantiate this claim, consistency among the sensor performance was also verified by measuring a baseline in the sensor’s response to a control load (here a light tactile load), which averaged 179 ± 60 MΩ across all sensors.

The results in Figs 7 and 8 raise various points that warrant further discussion. The observation of non-uniform pressure distributions and redistribution of pressure during use observed in this personalized prosthesis provides guidance for future studies on the optimization of comfort and function of other personalized wearable systems, such as exoskeletons with AHMIs. In particular, these measurements could inform the geometric and mechanical design of the device’s personalized interface (*e*.*g*., via mechanical property grading). For example, exposure to small pressures over prolonged time periods has been discussed as a potential mechanism of pressure ulcer formation [68], and thus, appears to be a central aspect of optimizing comfort and function of wearable systems. While anatomical customization is now becoming more widely discussed as an important design factor for obtaining optimal function from wearable devices other than prosthetic hands, such as passive-dynamic ankle-foot orthoses [69], we suggest that future work could also examine the role of anatomical customization on function of personalized prosthetic hands.

Beyond the pressure mapping studies described in Fig 8, the participant was also able to perform facile grasping tasks using the 3D printed personalized prosthetic hand that included grasping a 20 oz. plastic water bottle. A detailed assessment of the personalized prosthetic hand’s function was beyond the scope of this manuscript, which used a relatively facile flexion test to establish a low-cost methodology for personalizing and incorporating sensing and monitoring functionality to the anatomical interface. Future work could assess the effect of personalization on performance of the prosthetic hand beyond flexion tests. Assessment of 3D printed prosthetic hands, similar to the base model used here, has previously been examined [70]. In that work, a nine-hole pegboard test, box and block test, and hand strength test were used to assess hand function [70]. We refer the reader to a comprehensive discussion of hand function tests for evaluation of prosthetic hand performance [70, 71]. Briefly, the 3D printed personalized prosthetic hand examined in this work is classified as a body-powered prosthesis with grasping action that is actuated by flexion of the user’s wrist. Thus, while a number of tests have been developed for assessment of prosthetic hand function that involve various tasks (*e*.*g*., lifting and manipulation of objects) [70, 72, 73], we restricted our discussion to established methods for assessment of grasping function as the primary action of the prosthetic hand examined in this study. For example, Laliberte *et al*. used a task of grasping objects with different geometries and sizes, ranging from a tennis ball to a plug with different grasp types to assess the grasping function of their new kinematic design of the thumb [34]. Lee *et al*. used a similar test based on the task of picking up objects with the same size and measuring the number of objects being transferred within a certain time that provided the advantage of quantitative measurement and comparison of performance among different prostheses [70]. The Simple Test for Evaluating Hand Function (STEF) has also been commonly utilized for assessing the ability to pinch, grasp, and transfer objects [74].

## Conclusions

Here, we showed that the integration of 3D scanning with 3D printing enables the personalization of low-cost prosthetic hands with anatomically conformal electronic interfaces for children with amniotic band syndrome. Specifically, a 3D scanning-CAD process was used to create a personalized palm component associated with a widely used non-personalized prosthesis that enabled a form-fitting interface with the participant’s anatomy. We also reported a method for identification of optimal scanning parameters based on the use of local and global scan quality metrics. Personalization increased the tissue-prosthesis contact area, which enabled the integration of electronic components for pressure mapping through a low-cost conformal 3D printing format. We observed that the pressure distribution across the personalized tissue interface was non-uniform and became redistributed during wrist flexion. Overall, this work shows that the integration of 3D scanning and 3D printing processes offers the ability to rapidly design and fabricate low-cost personalized and anatomical wearable systems. It also suggests that 3D scanning and 3D printing create a useful computer-aided design and manufacturing framework for improving our understanding of the effect of personalization on wearable system comfort and function.

## Supporting information

S1 File 3D Printed Prosthetic Hand—PLoS One—SI.docx. Photograph of the 3D scanning experimental setup **(Figure A)**. Photographs and 3D models of the tested anatomical structures corresponding to Fig 3 of the main text. a) Left ear of an adult female. b) Adult porcine kidney. c) Limb malformation resulting from amniotic band syndrome for the participant of this study. d) Right hand of an adult male. e) Right foot of an adult male **(Figure B)**. 3D scanning data validating that personalization did not impede the prosthetic hand’s ability to create a grasping action corresponding to scans acquired in relaxed (left panel) and flexed (right panel) states actuated by the participant’s wrist flexion. We note that the low-density point cloud data was attributed to movement during scanning **(Figure C)**. Photographs of the participant wearing the 3D printed bionic prosthesis **(Figure D)**.
(DOCX)

## References

[1] Chua MCH, Chee Kong C, Bina R, Lau DDP, editors. Development of a patient specific artificial tracheal prosthesis: Design, mechanical behavior analysis and manufacturing. 35th Annual International Conference of the IEEE Engineering in Medicine and Biology Society (EMBC); 2013. 10.1109/EMBC.2013.6610978 24111165

[2] Murphy SV, Atala A. 3D bioprinting of tissues and organs. Nat Biotech. 2014;32(8):773–85. 10.1038/nbt.2958 25093879

[3] Chang JW, Park SA, Park J-K, Choi JW, Kim Y-S, Shin YS, et al Tissue-Engineered Tracheal Reconstruction Using Three-Dimensionally Printed Artificial Tracheal Graft: Preliminary Report. Artif Organs. 2014;38(6):E95–E105. 10.1111/aor.12310 24750044

[4] Johnson BN, Lancaster KZ, Zhen G, He J, Gupta MK, Kong YL, et al 3D Printed Anatomical Nerve Regeneration Pathways. Adv Funct Mater. 2015;25(39):6205–17. 10.1002/adfm.201501760 26924958 PMC4765385

[5] Schmalbruch H. Fiber composition of the rat sciatic nerve. Anat Rec. 1986;215(1):71–81. 10.1002/ar.1092150111 3706794

[6] Gough NR. Bioprinting Cartilage Scaffolds. Science Signaling. 2014;7(356):ec347.

[7] Temple JP, Hutton DL, Hung BP, Huri PY, Cook CA, Kondragunta R, et al Engineering anatomically shaped vascularized bone grafts with hASCs and 3D-printed PCL scaffolds. J Biomed Mater Res A. 2014;102(12):4317–25. 10.1002/jbm.a.35107 24510413

[8] Morrison RJ, Hollister SJ, Niedner MF, Mahani MG, Park AH, Mehta DK, et al Mitigation of tracheobronchomalacia with 3D-printed personalized medical devices in pediatric patients. Sci Transl Med. 2015;7(285):285ra64 10.1126/scitranslmed.3010825 25925683 PMC4495899

[9] Waran V, Narayanan V, Karuppiah R, Pancharatnam D, Chandran H, Raman R, et al Injecting Realism in Surgical Training—Initial Simulation Experience With Custom 3D Models. Journal of Surgical Education. 2014;71(2):193–7. 10.1016/j.jsurg.2013.08.010 24602709

[10] Rengier F, Mehndiratta A, von Tengg-Kobligk H, Zechmann CM, Unterhinninghofen R, Kauczor HU, et al 3D printing based on imaging data: review of medical applications. Int J CARS. 2010;5(4):335–41. 10.1007/s11548-010-0476-x 20467825

[11] Youssef RF, Spradling K, Yoon R, Dolan B, Chamberlin J, Okhunov Z, et al Applications of three-dimensional printing technology in urological practice. BJU International. 2015;116(5):697–702. 10.1111/bju.13183 26010346

[12] Tam MD, Laycock SD, Bell D, Chojnowski A. 3-D printout of a DICOM file to aid surgical planning in a 6 year old patient with a large scapular osteochondroma complicating congenital diaphyseal aclasia. Journal of Radiology Case Reports. 2012;6(1):31–7. 10.3941/jrcr.v6i1.889 22690278 PMC3370704

[13] Ibrahim D, Broilo TL, Heitz C, de Oliveira MG, de Oliveira HW, Nobre SMW, et al Dimensional error of selective laser sintering, three-dimensional printing and PolyJet™ models in the reproduction of mandibular anatomy. J Cranio Maxill Surg. 2009;37(3):167–73. 10.1016/j.jcms.2008.10.008 19056288

[14] Hochman JB, Kraut J, Kazmerik K, Unger BJ. Generation of a 3D Printed Temporal Bone Model with Internal Fidelity and Validation of the Mechanical Construct. Otolaryngol Head and Neck Surg. 2013;150:448–54. 24381017 10.1177/0194599813518008

[15] Forgacs G, Sun W. Biofabrication. First ed: Elsevier; 2013.

[16] Kong YL, Tamargo I, Kim H, Johnson BN, Gupta MK, Koh T-W, et al 3D printed quantum dot light-emitting diodes. Nano Lett. 2014;14:7017–23. 10.1021/nl5033292 25360485

[17] Singh M, Tong Y, Webster K, Cesewski E, Haring AP, Laheri S, et al 3D printed conformal microfluidics for isolation and profiling of biomarkers from whole organs. Lab on a Chip. 2017;17(15):2561–71. 10.1039/c7lc00468k 28632265

[18] Goyanes A, Det-Amornrat U, Wang J, Basit AW, Gaisford S. 3D scanning and 3D printing as innovative technologies for fabricating personalized topical drug delivery systems. J Control Release. 2016;234:41–8. 10.1016/j.jconrel.2016.05.034 27189134

[19] Shuang-Zhuang G, Kaiyan Q, Fanben M, Hyun PS, M C. M. 3D Printed Stretchable Tactile Sensors. Adv Mater. 2017;29(27):1701218. 10.1002/adma.201701218 PMC5509487 28474793

[20] Pathak VK, Singh AK, Sivadasan M, Singh N. Framework for automated GD&T inspection using 3D scanner. Journal of The Institution of Engineers (India): Series C. 2018;99(2):197–205.

[21] Kim M-K, Cheng JC, Sohn H, Chang C-C. A framework for dimensional and surface quality assessment of precast concrete elements using BIM and 3D laser scanning. Automation in Construction. 2015;49:225–38.

[22] Dawood A, Marti BM, Sauret-Jackson V, Darwood A. 3D printing in dentistry. British dental journal. 2015;219(11):521 10.1038/sj.bdj.2015.914 26657435

[23] Dastoorian R, Elhabashy AE, Tian W, Wells LJ, Camelio JA. Automated Surface Inspection Using 3D Point Cloud Data in Manufacturing: A Case Study. 2018;(51371):V003T02A36.

[24] Rusinkiewicz S, Hall-Holt O, Levoy M. Real-time 3D model acquisition. ACM T Graphic. 2002;21(3):438–46

[25] Boehnen C, Flynn P, editors. Accuracy of 3D scanning technologies in a face scanning scenario. Fifth International Conference on 3-D Digital Imaging and Modeling (3DIM'05); 2005.

[26] Apeagyei PR. Application of 3D body scanning technology to human measurement for clothing fit International Journal of Digital Content Technology and its Applications. 2010;4(7):58–68.

[27] Ziegler-Graham K, MacKenzie EJ, Ephraim PL, Travison TG, Brookmeyer R. Estimating the prevalence of limb loss in the United States: 2005 to 2050. Archives of physical medicine and rehabilitation. 2008;89(3):422–9. 10.1016/j.apmr.2007.11.005 18295618

[28] Graczyk EL, Resnik L, Schiefer MA, Schmitt MS, Tyler DJ. Home Use of a Neural-connected Sensory Prosthesis Provides the Functional and Psychosocial Experience of Having a Hand Again. Scientific reports. 2018;8(1):9866 10.1038/s41598-018-26952-x 29959334 PMC6026118

[29] ten Kate J, Smit G, Breedveld P. 3D-printed upper limb prostheses: a review. Disability and Rehabilitation: Assistive Technology. 2017;12(3):300–14. 10.1080/17483107.2016.1253117 28152642

[30] Zuniga JM, Peck J, Srivastava R, Katsavelis D, Carson A. An open source 3D-printed transitional hand prosthesis for children. JPO: Journal of Prosthetics and Orthotics. 2016;28(3):103–8.

[31] Dodziuk H. Applications of 3D printing in healthcare. Kardiochirurgia i torakochirurgia polska = Polish journal of cardio-thoracic surgery. 2016;13(3):283 10.5114/kitp.2016.62625 27785150 PMC5071603

[32] Phillips B, Zingalis G, Ritter S, Mehta K, editors. A review of current upper-limb prostheses for resource constrained settings. Global Humanitarian Technology Conference (GHTC), 2015 IEEE; 2015: IEEE.

[33] Bahari MS, Jaffar A, Low CY, Jaafar R, Roese K, Yussof H. Design and development of a multifingered prosthetic hand. International Journal of Social Robotics. 2012;4(1):59–66.

[34] Laliberté T, Baril M, Guay F, Gosselin C. Towards the design of a prosthetic underactuated hand. Mechanical Sciences. 2010;1(1):19–26.

[35] Kong YL, Gupta MK, Johnson BN, McAlpine MC. 3D printed bionic nanodevices. Nano Today. 2016;11:330–50. 10.1016/j.nantod.2016.04.007 27617026 PMC5016035

[36] Aszmann OC, Roche AD, Salminger S, Paternostro-Sluga T, Herceg M, Sturma A, et al Bionic reconstruction to restore hand function after brachial plexus injury: a case series of three patients. The Lancet. 2015;385(9983):2183–9. 10.1016/S0140-6736(14)61776-1 25724529

[37] Smith DG, Campbell KM. Prostheses for Children With Limb Differences: Amputee Coalition; 2009 [cited 2018 April 12th]. https://www.amputee-coalition.org/resources/prostheses-for-children/.

[38] Rushton DI. Amniotic Band Syndrom. British Medical Journal (Clinical research ed). 1983;286(6369):2. 10.1136/bmj.286.6369.919 PMC1547338 6403134

[39] Zuniga J, Katsavelis D, Peck J, Stollberg J, Petrykowski M, Carson A, et al Cyborg beast: a low-cost 3d-printed prosthetic hand for children with upper-limb differences. BMC Research Notes. 2015;8(1):10. 25601104 10.1186/s13104-015-0971-9 PMC4304188

[40] Wilson AB. Limb prosthetics. 5th ed Huntington, N.Y.: R. E. Krieger Pub. Co; 1976 95 p.

[41] Wilson AB. A primer on limb prosthetics. Springfield, Ill.: C.C. Thomas; 1998 151 p.

[42] Gretsch KF, Lather HD, Peddada KV, Deeken CR, Wall LB, Goldfarb CA. Development of novel 3D-printed robotic prosthetic for transradial amputees. Prosthetics and orthotics international. 2016;40(3):400–3. 10.1177/0309364615579317 25934422

[43] Patton JG. Developmental approach to pediatric prosthetic evaluation and training Comprehensive Management of the Upper-Limb Amputee: Springer; 1989 p. 137–49.

[44] Sypniewski BL. The child with terminal transverse partial hemimelia: a review of the literature on prosthetic management. Artificial limbs. 1972;16(1):20–50. 4567370

[45] Kuyper M, Breedijk M, Mulders A, Post M, Prevo A. Prosthetic management of children in The Netherlands with upper limb deficiencies. Prosthetics and orthotics international. 2001;25(3):228–34. 10.1080/03093640108726606 11860097

[46] Gupta MK, Meng F, Johnson BN, Kong YL, Tian L, Yeh Y-W, et al 3D Printed Programmable Release Capsules. Nano Lett. 2015;15(8):5321–9. 10.1021/acs.nanolett.5b01688 26042472 PMC4536147

[47] McAlpine MC, Sebastian-Mannoor M, Kong YL, Johnson BN, inventorsMulti-functional hybrid devices/structures using 3d printing. USA2016.

[48] Haring AP, Khan AU, Liu G, Johnson BN. 3D Printed Functionally Graded Plasmonic Constructs. Advanced Optical Materials. 2017;5(18):1700367.

[49] Cesewski E, Haring AP, Tong Y, Singh M, Thakur R, Laheri S, et al Additive manufacturing of three-dimensional (3D) microfluidic-based microelectromechanical systems (MEMS) for acoustofluidic applications. Lab Chip. 2018;18(14):2087–98. 10.1039/c8lc00427g 29897358 PMC6077993

[50] Mannoor MS, Jiang Z, James T, Kong YL, Malatesta KA, Soboyejo WO, et al 3D printed bionic ears. Nano Lett. 2013;13(6):2634–9. 10.1021/nl4007744 23635097 PMC3925752

[51] Guo S-Z, Qiu K, Meng F, Park SH, McAlpine MC. 3D Printed Stretchable Tactile Sensors. Adv Mater. 2017;29(27):1701218. 10.1002/adma.201701218 PMC5509487 28474793

[52] Cignoni P, Callieri M, Corsini M, Dellepiane M, Ganovelli F, Ranzuglia G, editors. Meshlab: an open-source mesh processing tool. Eurographics Italian chapter conference; 2008.

[53] Rusinkiewicz S, Levoy M, editors. Efficient variants of the ICP algorithm. 3-D Digital Imaging and Modeling, 2001 Proceedings Third International Conference on; 2001: IEEE.

[54] Pulli K, editor Multiview registration for large data sets. 3-D Digital Imaging and Modeling, 1999 Proceedings Second International Conference on; 1999: IEEE.

[55] Callieri M, Cignoni P, Ganovelli F, Montani C, Pingi P, Scopigno R, editors. VCLab’s Tools for 3D range data processing. VAST; 2003. 10.1109/mcg.2004.1274056 15387223

[56] Smits F. Measurement of sheet resistivities with the four-point probe. Bell System Technical Journal. 1958;37(3):711–8.

[57] Kong YL, Tamargo IA, Kim H, Johnson BN, Gupta MK, Koh T-W, et al 3D printed quantum dot light-emitting diodes. Nano letters. 2014;14(12):7017–23. 10.1021/nl5033292 25360485

[58] Johnson BN, Lancaster KZ, Zhen G, He J, Gupta MK, Kong YL, et al 3D printed anatomical nerve regeneration pathways. Advanced functional materials. 2015;25(39):6205–17. 10.1002/adfm.201501760 26924958 PMC4765385

[59] Shanmuganathan N, Maheswari MU, Anandkumar V, Padmanabhan T, Swarup S, Jibran AH. Aesthetic finger prosthesis. The Journal of Indian Prosthodontic Society. 2011;11(4):232–7. 10.1007/s13191-011-0074-9 23204732 PMC3205179

[60] Yogeswaran N, Tinku S, Khan S, Lorenzelli L, Vinciguerra V, Dahiya R, editors. Stretchable resistive pressure sensor based on CNT-PDMS nanocomposites. Ph D Research in Microelectronics and Electronics (PRIME), 2015 11th Conference on; 2015: IEEE.

[61] Lu N, Lu C, Yang S, Rogers J. Highly sensitive skin-mountable strain gauges based entirely on elastomers. Advanced Functional Materials. 2012;22(19):4044–50.

[62] Sepúlveda A, de Villoria RG, Viana J, Pontes A, Wardle B, Rocha LA. Full elastic constitutive relation of non-isotropic aligned-CNT/PDMS flexible nanocomposites. Nanoscale. 2013;5(11):4847–54. 10.1039/c3nr00753g 23616092

[63] Pang C, Lee G-Y, Kim T-i, Kim SM, Kim HN, Ahn S-H, et al A flexible and highly sensitive strain-gauge sensor using reversible interlocking of nanofibres. Nature materials. 2012;11(9):795 10.1038/nmat3380 22842511

[64] Dahiya RS, Metta G, Valle M, Sandini G. Tactile sensing—from humans to humanoids. IEEE transactions on robotics. 2010;26(1):1–20.

[65] Schwartz G, Tee BC-K, Mei J, Appleton AL, Kim DH, Wang H, et al Flexible polymer transistors with high pressure sensitivity for application in electronic skin and health monitoring. Nature communications. 2013;4:1859 10.1038/ncomms2832 23673644

[66] Ahn BY, Walker SB, Slimmer SC, Russo A, Gupta A, Kranz S, et al Planar and three-dimensional printing of conductive inks. Journal of visualized experiments: JoVE. 2011;(58). 10.3791/3189 PMC3346051 22214978

[67] Kong K, Mariatti M, Rashid A, Busfield J. Effect of processing methods and functional groups on the properties of multi-walled carbon nanotube filled poly (dimethyl siloxane) composites. Polymer bulletin. 2012;69(8):937–53.

[68] Bhattacharya S, Mishra R. Pressure ulcers: current understanding and newer modalities of treatment. Indian Journal of Plastic Surgery: Official Publication of the Association of Plastic Surgeons of India. 2015;48(1):4. 10.4103/0970-0358.155260 PMC4413488 25991879

[69] Schrank ES. Dimensional accuracy of ankle-foot orthoses constructed by rapid customization and manufacturing framework. Journal of rehabilitation research and development. 2011;48(1):31 21328161 10.1682/jrrd.2009.12.0195

[70] Lee KH, Bin H, Kim K, Ahn SY, Kim B-O, Bok S-K. Hand Functions of Myoelectric and 3D-Printed Pressure-Sensored Prosthetics: A Comparative Study. Annals of rehabilitation medicine. 2017;41(5):875–80. 10.5535/arm.2017.41.5.875 29201828 PMC5698676

[71] Light C, Chappell P, Kyberd P, Ellis B. A critical review of functionality assessment in natural and prosthetic hands. British Journal of Occupational Therapy. 1999;62(1):7–12.

[72] Light CM, Chappell PH, Kyberd PJ. Establishing a standardized clinical assessment tool of pathologic and prosthetic hand function: normative data, reliability, and validity. Archives of physical medicine and rehabilitation. 2002;83(6):776–83. 12048655 10.1053/apmr.2002.32737

[73] Kyberd PJ, Murgia A, Gasson M, Tjerks T, Metcalf C, Chappell PH, et al Case studies to demonstrate the range of applications of the Southampton Hand Assessment Procedure. British Journal of Occupational Therapy. 2009;72(5):212–8.

[74] Kita K, Otaka Y, Takeda K, Sakata S, Ushiba J, Kondo K, et al A pilot study of sensory feedback by transcutaneous electrical nerve stimulation to improve manipulation deficit caused by severe sensory loss after stroke. Journal of neuroengineering and rehabilitation. 2013;10(1):55. 23764012 10.1186/1743-0003-10-55 PMC3701472

