# Supplementary material for: Low-cost sensor-integrated 3D-printed personalized prosthetic hands for children with amniotic band syndrome: A case study in sensing pressure distribution on an anatomical human-machine interface (AHMI) using 3D-printed conformal electrode arrays
Source: PLoS One. 2019 Mar 28;14(3):e0214120. doi: 10.1371/journal.pone.0214120 (PMC6438526; doi:10.1371/journal.pone.0214120)
Supplement: S1 File — Photograph of the 3D scanning experimental setup (Figure A). Photographs and 3D models of the tested anatomical structures corresponding to Fig 3 of the main text. a) Left ear of an adult female. b) Adult porcine kidney. c) Limb malformation resulting from amniotic band syndrome for the participant of this study. d) Right hand of an adult male. e) Right foot of an adult male (Figure B). 3D scanning data validating that personalization did not impede the prosthetic hand’s ability to create a grasping action corresponding to scans acquired in relaxed (left panel) and flexed (right panel) states actuated by the participant’s wrist flexion. We note that the low-density point cloud data was attributed to movement during scanning (Figure C). Photographs of the participant wearing the 3D printed bionic prosthesis (Figure D). (DOCX) [file pone.0214120.s001.docx]

**Low-cost Sensor-integrated 3D Printed Personalized Prosthetic Hands for Children with Amniotic Band Syndrome: A Case Study in Sensing Pressure Distribution on an Anatomical Human-Machine Interface (AHMI) using 3D Printed Conformal Electrode Arrays**

*Yuxin Tong,*^1^ *Ezgi Kucukdeger,*^1^ *Justin Halper,*^1^ *Ellen Cesewski,*^2^ *Elena Karakozoff,*^1^ *Alexander P. Haring,*^3^ *David McIlvain,*^1^ *Manjot Singh,*^1^ *Nikita Khandelwal,*^1^ *Alex Meholic,*^1^ *Sahil Laheri,*^4^ *Akshay Sharma,*^5^ *and Blake N. Johnson*^1,2-4^*^*^*

^1^ Department of Industrial and Systems Engineering, Virginia Tech, Blacksburg, VA 24061 USA

^2^ Department of Materials Science and Engineering, Virginia Tech, Blacksburg, VA 24061 USA

^3^ Macromolecules Innovation Institute, Virginia Tech, Blacksburg, VA 24061 USA

^4^ School of Neuroscience, Virginia Tech, Blacksburg, VA 24061 USA

^5^ School of Architecture + Design, Virginia Tech, Blacksburg, VA 24061 USA

**Keywords:** 3D Printing; Personalized Prosthetics; Additive Manufacturing; Conformal 3D Printing; Bionics; Wearable Systems

*Corresponding Author - E-mail: [bnj@vt.edu](mailto:bnj@vt.edu); Phone: 540-231-0755

This supporting information contains: 1) a photograph of the 3D scanning experimental setup; 2) photographs and digital models of the different anatomical structures discussed within the main text; 3) 3D scanning data validating the prosthetic hand’s body-powered grasping action post-personalization; and 4) photographs of participant wearing the bionic prosthesis.


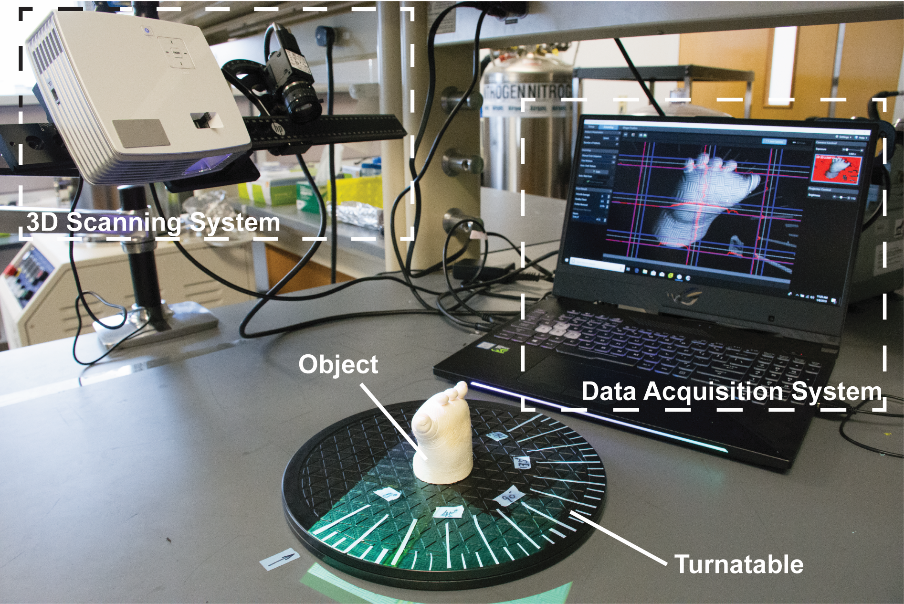


**Figure A.**


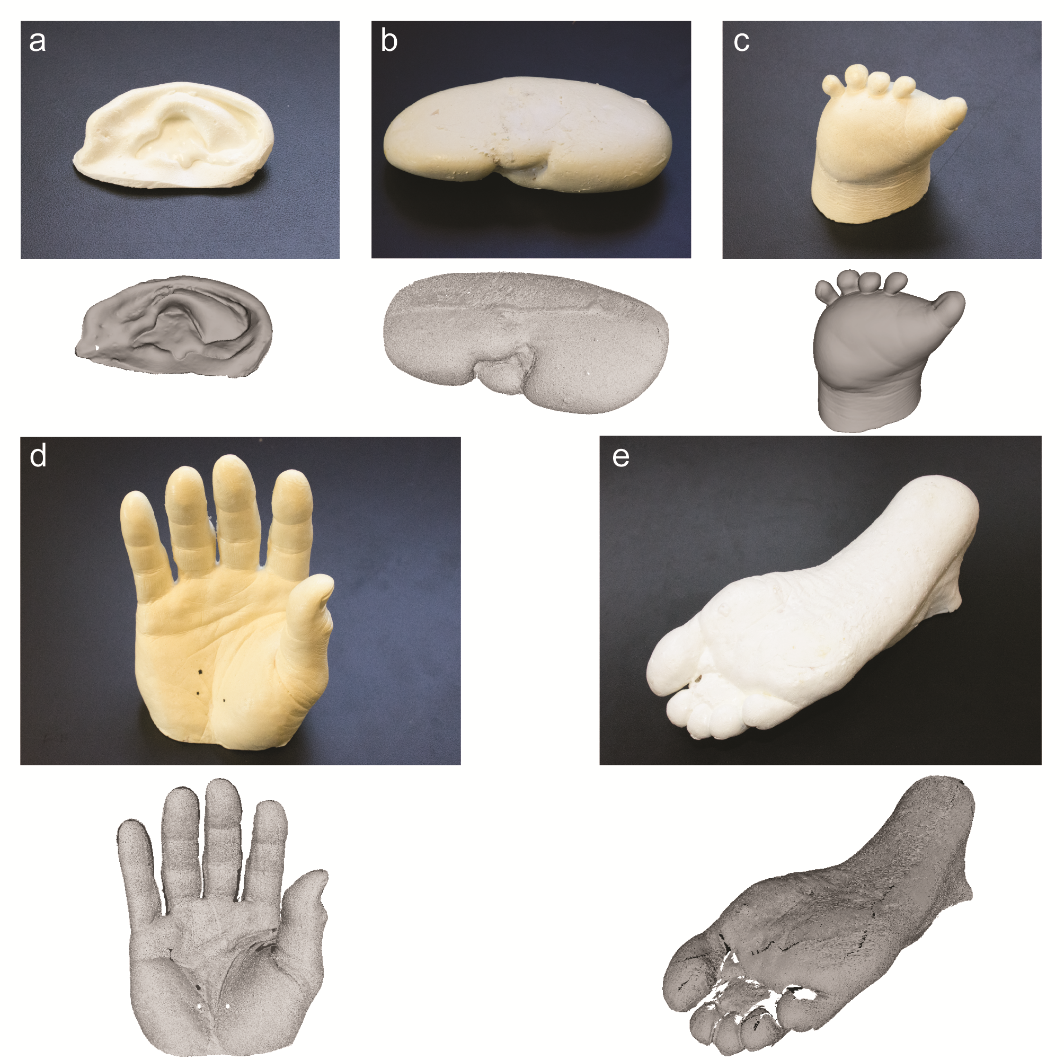


**Figure B.**

**
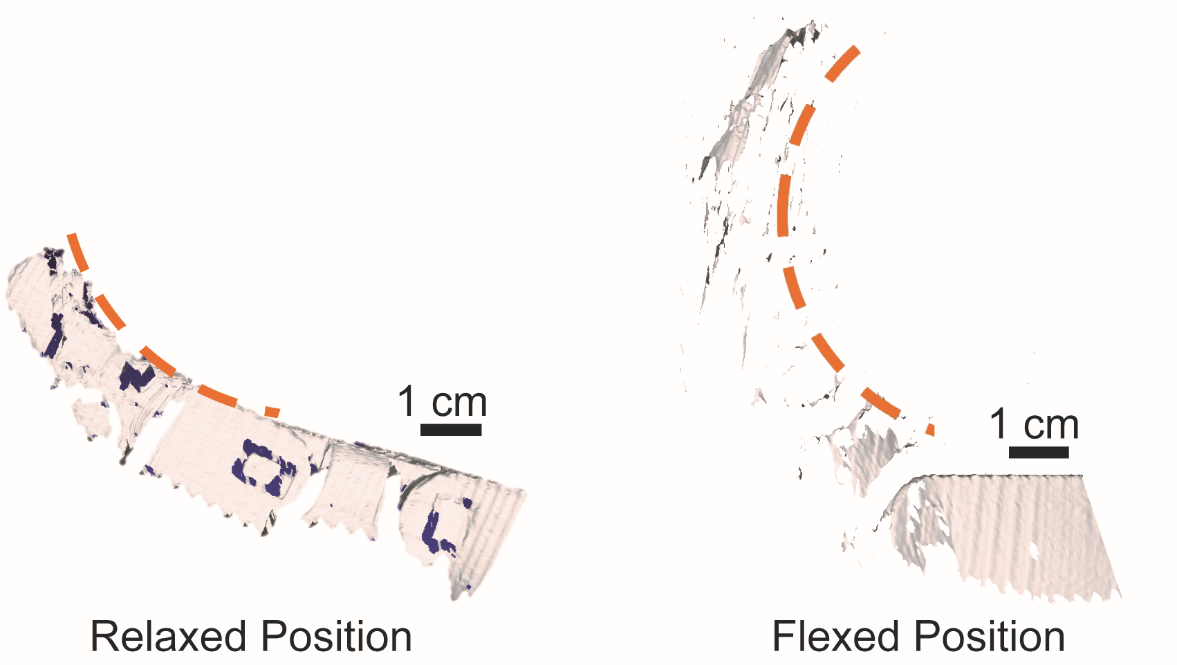
**

**Figure C.**


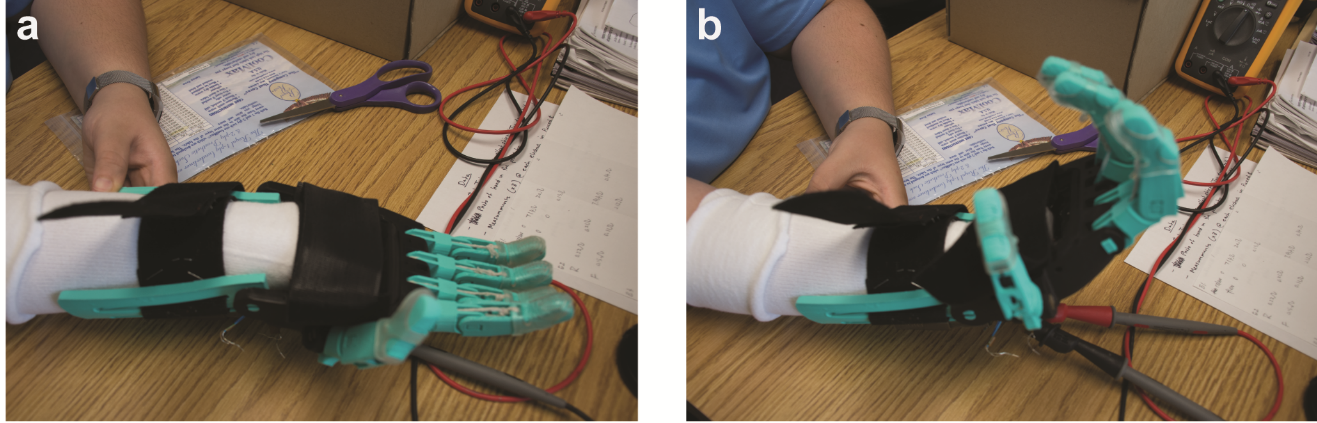


**Figure D.**
